# Tumeur pseudopapillaire et solide du pancréas: à propos d'un cas et revue de la literature

**DOI:** 10.11604/pamj.2016.24.104.8301

**Published:** 2016-05-31

**Authors:** Nabil Jakhlal, Noureddine Njoumi, Hafid Hachi, Abdesslam Bougtab

**Affiliations:** 1 Service de Chirurgie K, Institut National d'Oncologie de Rabat, Maroc

**Keywords:** Pancréas, tumeur pseudopapillaire et solide, Frantz, résection chirurgicale, Pancreas, solid pseudopapillary tumour, Frantz, surgical resection

## Abstract

Les tumeurs pseudopapillaires et solides du pancréas sont des tumeurs rares, d’étiopathogénie encore incertaine, et surviennent surtout chez la jeune femme. Leur pronostic reste bon surtout après exérèse complète. Nous rapportons une nouvelle observation d'une jeune femme, révélée par des douleurs abdominales. Le diagnostic est porté sur l’étude histologique associée à l'immuno-histochimie de la pièce de spléno-pancréatectomie caudale.

## Abstract (en)

Solid pseudopapillary tumours of the pancreas are rare tumours of uncertain etiology which mostly affects young women. Their prognosis is good especially after complete resection. We report a new case of a young woman; abdominal pain revealed solid pseudopapillary tumour. The diagnosis is based on histological examination associated with immunohistochemistry of the piece of resection removed with caudal splenopancreatectomy.

## Introduction

Les tumeurs pseudopapillaires et solides du pancréas (TPPSP) sont rares. Elles représentent moins de 2% des cancers pancréatiques. Elles touchent essentiellement les femmes jeunes. Leur étiopathogénie reste incertaine. Elles sont caractérisées par un polymorphisme clinique et radiologique ce qui rend leur diagnostic difficile. Le seul traitement garant d'une survie prolongée est la résection chirurgicale. Leur pronostic est excellent. Le but de ce travail est de rapporter une nouvelle observation et de rappeler les principales données concernant ces tumeurs disponibles dans la littérature.

## Patient et observation

**Figure 1:**
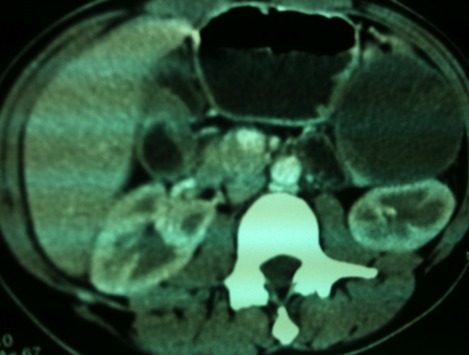
Image scannographique montrant une masse bien limitée, hétérogène de la queue du pancréas

Une patiente âgée de 21 ans, sans antécédents, était admise pour des douleurs de l'hypochondre gauche d'installation progressive, à type de pesanteur, sans signes accompagnateurs. L'examen physique était sans particularités. L’échographie abdominale montrait une masse tissulaire et multi-kystique entre la rate et la queue du pancréas. La TDM abdominale avait conclu à une masse kystique, bien limitée, finement cloisonnée de la queue du pancréas, mesurant 7cm (Figure 1). Le traitement chirurgical consistait en une spléno-pancréactomie caudale emportant la masse kystique avec splénectomie partielle. L’étude morphologique et l'immuno-histochmie (les anticorps positifs sont: anti-CD10, anti-vimentine, anti-NSE, anti-CD56, anti-RP et anti-synaptophysine) sont revenues en faveur d'une TPPSP avec résection complète. Les suites opératoires étaient simples. Avec un recul de 4 ans, la patiente est en bon état général, sans récidive clinique ni scannographique.

## Discussion

La tumeur pseudo papillaire et solide du pancréas (TPPSP) a été décrite pour la première fois par Frantz en 1959 [1]. Il s'agit d'une tumeur rare qui représente moins de 2% des tumeurs pancréatiques exocrines et moins de 5% des tumeurs kystiques du pancréas [2]. Elle touche généralement les jeunes femmes avec un âge moyen de 28 et un sex-ratio de 10:1 [3]. Cependant, des rares cas sporadiques chez les hommes et les personnes âgées ont également été signalés. Cette tumeur atteint aussi bien la tête, le corps ou la queue du pancréas, avec néanmoins une prédominance dans la région corporéo-caudale (64% des cas) [4]. De rares cas de localisations extrapancréatiques sont aussi décrits (1%) à savoir rétropéritonéale, duodénale, mésocolique, hépatique [4, 5]. Les circonstances de découverte sont très variables et peu spécifiques: il peut s'agir d'une découverte fortuite lors d'un examen d'imagerie réalisé pour une autre raison, ou lors de l'apparition d'une masse abdominale palpable, ou encore par des douleurs abdominales aspécifiques. Parfois la tumeur, en augmentant de taille, entraîne des signes de compression des structures digestives, biliaires ou vasculaires de voisinage [6]. Elle peut être révélée dans les suites d'un traumatisme abdominal (3%) ou à l'occasion d'une complication à type d'une rupture ou d'une hémorragie intra tumorale [7]. Les examens complémentaires montrent généralement une masse complexe bien encapsulée avec les deux composantes solides et kystiques. L'aspect échographique de la tumeur varie en fonction de l'importance des zones kystiques. Mais dans presque tous les cas, la TPPSP se présente comme une masse kystique bien limitée, à contours réguliers peu ou pas vascularisée, à contenu hétérogène et sans cloisons intérieurs [5]. La tomodensitométrie abdominale montre une masse bien limitée, hétérogène, solide et kystique, se rehaussant peu ou partiellement en périphérie après injection du produit de contraste [8]. L'IRM constitue le meilleur moyen pour obtenir des informations sur l'hémorragie au sein de la lésion par une imagerie multi-plan [9]. Elle permet aussi de mettre en évidence la capsule fibreuse et de différencier les composantes solides et kystiques intra-tumorales [10]. L’écho-endoscopie apporte une précision plus importante sur les petites lésions pancréatiques de diamètre inférieur à 2 cm qui sont habituellement indétectables par les techniques d'imagerie usuelles (échographie, TDM, IRM) [11]. La cytoponction percutanée écho-guidée peuvent aider à distinguer la TPPSP d'autres tumeurs pancréatiques. Cependant, la biopsie percutanée est associée à un risque non négligeable de dissémination tumorale sur son trajet et de complications comme le saignement, fistule pancréatique et fistule biliaire [12]. Les données de la TDM ou IRM combinées avec l’âge et le sexe devraient être suffisantes pour indiquer une intervention chirurgicale, et la biopsie préopératoire devrait être effectuée lorsque le diagnostic radiologique n'est pas assez clair [13]. Sur le plan histologique, La tumeur est constituée de plages solides périphériques et de structures papillaires centrales. Les cellules tumorales sont monomorphes, de petite taille, cuboïdes ou polygonales et souvent agencées autour de septa fibro-vasculaires. Les mitoses et les atypies cytonucléaires sont exceptionnelles. On peut trouver des amas d'histiocytes spumeux et des cellules géantes autour de cristaux de cholestérol. Le stroma est habituellement de type endocrine, riche en capillaires sanguins. Les critères anatomo-pathologiques de malignité sont retrouvés dans seulement 10 à 15% des cas (envahissement des structures adjacentes, emboles vasculaires, invasion périnerveuse et métastases ganglionnaires ou à distance); dans ces cas, la TPPSP est classée carcinome pseudo papillaire et solide [14]. Le profil immuno-histochimique de la TPPSP est variable. Habituellement, les cellules tumorales sont marquées par les anticorps anti-CD 10, alpha-1- antitrypsine, vimentine, NSE, E-cadérine et bêtacaténine. On note aussi un marquage à l'anticorps anti-progestérone [15]. L'immuno-marquage positif des cellules tumorales pour certains marqueurs endocrines peut attester d'une certaine différenciation endocrine [16]. Le seul traitement curatif est chirurgical, dont le choix de la méthode opératoire dépend de la taille, la localisation tumorale et d'un éventuel envahissement des organes adjacents. Il consiste en une pancréatectomie gauche avec si possible conservation de la rate, une duodéno-pancréatectomie céphalique, une pancréatectomie partielle, voire totale [17]. Cependant, le faible degré de malignité de ces tumeurs et la présence d'une capsule fibreuse dense ont conduit plusieurs chirurgiens à tenter la simple énucléation [18], surtout en absence d'envahissement capsulaire [19]. L'exérèse doit être étendue en cas d'invasion des organes de voisinage, et d’éventuels nodules de carcinose péritonéale doivent être réséqués [20]. L'existence d'un envahissement des veines portes ou mésentériques ne doit pas contre-indiquer un geste à visée curative, des cas de résection portale ou mésentérique supérieure ayant été rapportés avec une survie prolongée [5]. Les lésions métastatiques associées doivent être réséquées avec un risque acceptable, et les récidives tumorales doivent bénéficier d'une tentative d'exérèse chirurgicale [8]. Le curage ganglionnaire reste controversé. La place d'une chimiothérapie ou d'une radiothérapie adjuvante est discutable [8]. Il en est de même pour l'hormonothérapie, utilisée en raison de la positivité de certaines tumeurs aux récepteurs à la progestérone, mais sans efficacité réelle [17]. Le pronostic des TPPSP est bon. Le taux de récidive est de 10 à 15% [8, 20]. Des cas de survie prolongée ont été rapportés même en présence de métastases hépatiques ou péritonéales ou en cas de chirurgie incomplète [20].

## Conclusion

La tumeur pseudo-papillaire et solide du pancréas est une tumeur rare. Son diagnostic repose sur l'histologie couplée à l'immuno-histochimie. Le bon pronostic de ces tumeurs justifie une attitude chirurgicale radicale, y compris pour les tumeurs métastatiques.
